# Characterization of Arbuscular Mycorrhizal Fungus Communities of *Aquilaria crassna* and *Tectona grandis* Roots and Soils in Thailand Plantations

**DOI:** 10.1371/journal.pone.0112591

**Published:** 2014-11-14

**Authors:** Amornrat Chaiyasen, J. Peter W. Young, Neung Teaumroong, Paiboolya Gavinlertvatana, Saisamorn Lumyong

**Affiliations:** 1 Department of Biology, Faculty of Science, Chiang Mai University, Chiang Mai, Thailand; 2 Department of Biology, University of York, York, United Kingdom; 3 Schoool of Biotechnology, Institute of Agricultural Technology, Suranaree University of Technology, Nakhon Ratchasima, Thailand; 4 Thai Orchid Labs Co. Ltd., Khannayao, Bangkok, Thailand; Nanjing Agricultural University, China

## Abstract

*Aquilaria crassna* Pierre ex Lec. and *Tectona grandis* Linn.f. are sources of resin-suffused agarwood and teak timber, respectively. This study investigated arbuscular mycorrhizal (AM) fungus community structure in roots and rhizosphere soils of *A. crassna* and *T. grandis* from plantations in Thailand to understand whether AM fungal communities present in roots and rhizosphere soils vary with host plant species and study sites. Terminal restriction fragment length polymorphism complemented with clone libraries revealed that AM fungal community composition in *A. crassna* and *T. grandis* were similar. A total of 38 distinct terminal restriction fragments (TRFs) were found, 31 of which were shared between *A. crassna* and *T. grandis*. AM fungal communities in *T*. *grandis* samples from different sites were similar, as were those in *A. crassna*. The estimated average minimum numbers of AM fungal taxa per sample in roots and soils of *T. grandis* were at least 1.89 vs. 2.55, respectively, and those of *A. crassna* were 2.85 vs. 2.33 respectively. The TRFs were attributed to Claroideoglomeraceae, Diversisporaceae, Gigasporaceae and Glomeraceae. The Glomeraceae were found to be common in all study sites. Specific AM taxa in roots and soils of *T. grandis* and *A. crassna* were not affected by host plant species and sample source (root vs. soil) but affected by collecting site. Future inoculum production and utilization efforts can be directed toward the identified symbiotic associates of these valuable tree species to enhance reforestation efforts.

## Introduction

Tropical forests are disappearing at the rate of 13.5 million hectares each year owing to logging, burning and clearing for agriculture and shifting cultivation [1]. At present, managed woodlands are required for timber and non-timber products in many countries. *Aquilaria crassna* Pierre ex Lec. (agarwood) and *Tectona grandis* Linn.f. (teak) are perennial plants that are used extensively to provide aromatic resin-infused wood products [2] and good quality teak wood products [3], respectively. The depletion of wild trees from indiscriminate cutting of *Aquilaria* species has resulted in the trees being listed and protected as endangered species. All *Aquilaria* species were listed in Appendix II of the Convention on International Trade in Endangered Species of Wild Fauna and Flora in 2005 [4]; however, a number of countries have outstanding reservations regarding that listing. Plantlets of *A. crassna* and *T. grandis* are produced in Thailand for domestic and foreign markets such as Jamaica, Guatemala, Mozambique, Sri Lanka, Indonesia, Laos, Malaysia, and Australia. Most *T. grandis* plantations in Thailand are planted in the northern provinces such as Chiang Mai, Chaing Rai and Phetchabun, while *A. crassna* plantations are mostly in eastern (Rayong, Trat and Chanthaburi provinces) and central (Nakhon-Nayok) Thailand.

Arbuscular mycorrhizal fungi (AMF) are soil fungi in the phylum Glomeromycota [5] that are mutualistically associated with roots of a wide spectrum of tropical and temperate tree species [6]. AM fungi have major effects on plant growth such as enhance the nutrient uptake by plant roots (especially phosphorus), particularly in low fertility soils [7], [8], protected plant against drought stress [9], [10], protect plant from soil-borne plant pathogenic infection [11], and improve soil aggregate stability through the action of mycelia and glomalin [12], [13], [14]. AMF inocula applied to plantlets and plant seedlings increased growth during early tree establishment in the field [2], [15], [16]. AM fungi have been used to inoculate and enhance growth of *T. grandis* [3], [17] and *Aquilaria* spp. [2], [18] prior to planting out. Therefore, studying the AM fungal communities of these plants in the field should aid plantation establishment and reforestation efforts. Information about the diversity of AM fungi associated with both plants has been reported mostly from natural forests in India [19], [20], [21], [22], [23] and only in *T. grandis* from Thailand [24]. These studies characterized communities based upon spore morphology. However, there are no reports of AM fungal communities of either tree using molecular tools. Identification of AM fungi based on spore morphology inevitably has some limitations, e.g. omission of AM fungi that did not produce spores during the sampling period and inability to identify the AM fungi within the roots.

PCR-based methods have been widely used in AM fungal community studies. Various studies have designed sets of specific primers for AM fungi [25], [26], [27] to facilitate rapid detection and identification directly from field-grown plant roots. Previously, Terminal restriction fragment length polymorphism (T-RFLP) has been used to study the AM fungi community in roots of arable crops [28], perennial herbs [29], herbaceous flowering plants [30], grass species [31], [32], grass species with herbaceous flowering plants [33], [34], and temperate deciduous trees [35]. Populations of AM fungi have been well studied in a number of ecosystems around the world, but there is scant information available for tropical forests and plantations of tropical and sub-tropical species.

This study provides the first molecular community analysis of AM fungi associated with field-collected roots and rhizosphere soils of the tropical trees *A. crassna* and *T. grandis*, and is part of a long term goal of optimizing AM fungus inoculation strategies to enhance reforestation efforts with these trees. It also provides an early insight into the biodiversity of AM fungi in Thailand to test the hypothesis that differences in AM fungal communities present in the roots and rhizosphere soils are determined by collecting sites, host plant species, and local environmental factors.

## Materials and Methods

### Ethics Statement

No specific permits were required to carry out research in the plantations: Chiang Mai (99°15' E, 18°58' N), Chiang Rai (99°29′ E/19°14′ N), Nakhon-Nayok (101°16′ E, 14°9′ N), Phetchabun (100°47' E, 16°2' N) and Thai Orchids Lab Ltd. (101°7′ E, 14°16′ N). The field studies did not involve endangered or protected species in Thailand. *Aquilaria crassna* is defined to be the forbidden forest item in only the forest area as the Forest Act. Therefore, the *A. crassna* planting and deforestation in the land of ownership is legal. All *A. crassna* samples were obtained from privately-owned plantations and are therefore not subject to the restrictions of the Forest Act of Thailand. Permission to sample the *T. grandis* and *A. crassna* were granted by the landowner.

### Study sites and sampling

Rhizosphere soils and roots were sampled from plantations of *T. grandis* and *A. crassna* in four provinces of Thailand (Table 1). Two sampling sites were located in Chiang Mai and Chiang Rai provinces in the northern region. These sites are monocultures of *T. grandis* planted at 2 m spacings and left to grow naturally with accumulated leaf litter and negligible understory perennial plants. Only roots attached to the main roots of *T. grandis* were sampled. At the sites in the central region; Nakhon-Nayok and Thai Orchids Lab Ltd., Nakhon-Nayok province, and in the northern region; Phetchabun province, *T. grandis* and *A. crassna* were planted alternately 2 m apart at Thai Orchids Lab Ltd. and Phetchabun. At both sites, weeds were controlled by ploughing and herbicide treatment. Thus, both species were planted without any above-ground vegetation, while in Nakhon-Nayok site, *A. crassna* was left to grow naturally. Paired soil and root samples from each plant species were randomly collected from 3 locations per site at 0–15 cm depth within 50 m^2^ and taken to the laboratory. All collections were carried out in July 2010. Root fragments were washed free of soil and air dried on tissue paper. Root fragments and soil samples were stored frozen at −20°C until further analysis.

**Table 1:** Chemical characteristic of soils (mean value ± SEM) in wet season (July 2010) which soils and roots were sampled.

Study plot	Soil pH^a^	Electrical conductivity a	Soil organic carbon (%)^a^	Total inorganic N (g kg^−1^ soil)^a^	Available P (mg kg^−1^ soil)^a^	Exchangeable K (mg kg^−1^ soil)^a^	Agricultural management
Phetchabun (PB)	5.77±0.25ab	0.18±0.02a	4.26±0.45ab	222±15a	156±72ab	449±163a	plowing, organic fertilizer, herbicide
Thai Orchid Lab (TO)	6.68± 0.28a	0.14±0.01a	4.15±1.48ab	188±57a	370±158a	296±81a	plowing, organic fertilizer, herbicide
Chiang Mai (CM)	5.70± 0.26bc	0.11±0.05a	6.10 ±0.87a	210±68a	171±133ab	284±10a	No management
Chiang Rai (CR)	5.23±0.11c	0.10±0.04a	2.83±0.25b	140±17a	24±3b	243±28a	No management
Nakhon Nayok (NN)	6.19±0.14ab	0.21±0.07a	3.08±0.66b	200±8a	149±63ab	347±78a	No management

a Means of three observations. Means in the same column followed by the same letter are not significantly different (α = 0.05).

### Soil analyses

Soil pH and electrical conductivity (EC) were determined in a 1∶1 soil: water slurry by direct measurement with pH-meter (Waterproof EC Testr, EUTECH instruments). Available phosphorus was measured employing the Bray II method [36]. Total inorganic nitrogen, exchangeable potassium and soil organic carbon were quantified following the methods of soil analysis outlined in Sparks et al. [37].

### Molecular analysis

Three replicate rhizosphere soil and root samples from each plant species were used to represent each site of collection. DNA was extracted from rhizophere soils and roots using the PowerSoil DNA isolation kit (MoBio Laboratories, CA) and Nucleospin Plant II (Macherey-Nagel GmbH & Co. KG, Düren), respectively according to the manufacturers' protocols. DNAs were amplified separately by nested PCR and then 20 µl of each of the three replicates from each sampling site were pooled and purified before restriction digestion [38]. The first round of AMF-specific PCR amplification was performed using the unlabelled primers AML1 and AML2 [26] with 30 cycles. In this first PCR, 40 µl reactions were carried out and each mixture contained 10 pmol of each primer, 1 unit of Taq polymerase (Promega) and 25 mM of each dNTP (Invitrogen) in manufacturer's reaction buffer (Promega). PCR was performed on a PTC100 thermocycler (MJ Research) with an initial denaturation at 94°C for 15 min, followed by 30 cycles of denaturation at 94°C for 30 s, annealing at 57°C for 45 s, extension at 72°C for 45 s, followed by a final extension of 72°C for 5 min. PCR products were visualized on a 1% agarose gel containing 0.1× SybrSafe (Invitrogen). The second round primers, 0.5 unit of Taq polymerase (Promega) and 20 pmol of HEX-labeled NS31 and FAM-labeled AML3 were added directly into 24 µl of each resulting product. Second-round PCR was conducted with 5 additional cycles using the same PCR conditions as the first PCR. The PCR products were purified using the QIAquick PCR purification kit (Qiagen). The purified PCR products were digested separately with the selected restriction enzymes, HinfI, Hsp92II and MboI (Promega) [31], [39] for 3 h at 37°C. Digested products were purified as mentioned above. Terminal restriction fragment (TRF) sizes from each sample were determined using the ABI PRISM 3130 Genetic Analyzer System (Applied Biosystems) with GeneScan LIZ-600 (Applied Biosystems) as internal size standards. The GeneMapper software (Applied Biosystems) was used for the analysis of fragment data. To reduce data noise, only fragments containing intensity above a baseline threshold (50 fluorescence units) were recorded. Relative peak heights were calculated and fragments with an average relative abundance <5% were excluded from further analysis.

### Screening and DNA sequence analysis

The remainders of the first PCR products were combined and purified using the QIAquick PCR purification kit (Qiagen). Purified DNA was cloned into pGEM-T Easy Vector (Promega) and transformed into *Escherichia coli* JM109. One hundred transformants were selected randomly and their insertion checked by PCR using the same primers, AML1 and AML2. The amplified DNAs were digested by the restriction enzymes HinfI and Hsp92II separately. One clone of each RFLP type was screened and sequenced using sequencing primers SP6 and T7 on an ABI PRISM 3130 Genetic Analyzer System (Applied Biosystems). Sequences were trimmed to the NS31-AML3 region and virtually digested with the restriction enzymes HinfI, Hsp92II, and MboI using an online restriction mapping website (RestrictionMapper).

### Phylogenetic analysis

Phylogenetic analysis was carried out on the sequences obtained in this study and those corresponding to the closest matches from Genbank, as well as sequences from cultured AMF taxa including representatives of the major groups of Glomeromycota from GenBank. All sequences obtained from this study were aligned by ClustalX using the BioEdit sequence alignment editor [40] along with 28 AMF sequences from GenBank. The aligned SSU rRNA dataset was trimmed to 450 bp by excluding the terminal primer sequences. A neighbour-joining (NJ) phylogeny was constructed using PAUP*4b10 [41] with the Kimura 2-parameter model and 1000 bootstraps. The nucleotide sequences of the clones retrieved in this study have been deposited in GenBank (accession numbers JQ8643324-JQ864355).

### Statistical analysis

The total number of TRFs was used as an AM fungal community diversity measurement [31]. The main and interaction effects of collecting sites, host plant species and sample source (root vs. soil) on number of TRFs using three restriction enzymes were tested with two-way factorial ANOVA using SPSS 11.5 for Windows (SPSS Inc., Chicago, IL, USA). Jaccard similarity coefficients were calculated for the T-RFLP patterns of root and soil samples of both plants, which were clustered by the unweighted pair-group average (UPGMA) algorithm with 1000 bootstrap replicates to obtain confidence estimates. These calculations were performed using FreeTree [42] and the results displayed using TreeView [43].

## Results

### Soil analyses and correlation with TRFs

Chemical characteristics of soil varied among sites (Table 1). Soil pH values ranged from 5.23 to 6.68. No significant different was observed in electrical conductivity, exchangeable potassium, and total inorganic nitrogen. Available phosphorus in soils tended to be highest at the Thai Orchid Lab site (370 mg kg^−1^soil) and differed significantly from the Chiang Rai site (24 mg kg^−1^soil). Soil organic carbon was highest at the Chiang Mai site (6.10%) and differed significantly from the Chiang Rai and Nakhon Nayok sites. Pearson correlation analysis between the soil properties measured and TRFs showed that TRFs were positively correlated with available phosphorus, organic matter, and pH (Table S1).

### AM fungal community of root and soil samples from *T. grandis* and *A. crassna*

**Figure 1:**
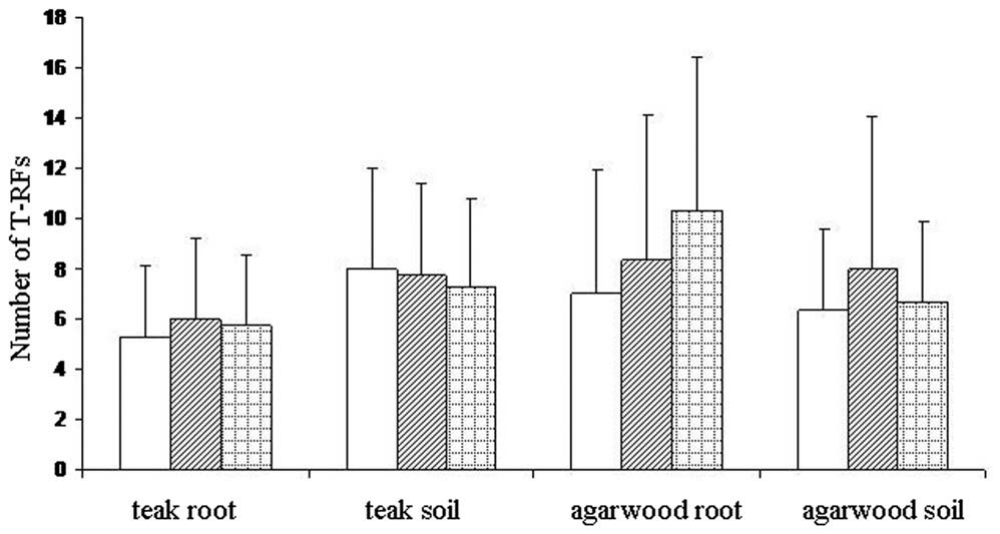
Effects of host plant, *Aquilaria crassna* (agarwood) and *Tectona grandis* (teak), and source of samples (root and soil) on mean number of terminal restriction fragments (TRFs) per sample using three restriction enzymes *MboI* (open bars), *HinfI* (hatched bars) and *Hsp92II* (cross-hatched bars). Values are mean ± SEM (n = 4 for teak and n = 3 for agarwood).

**Figure 2:**
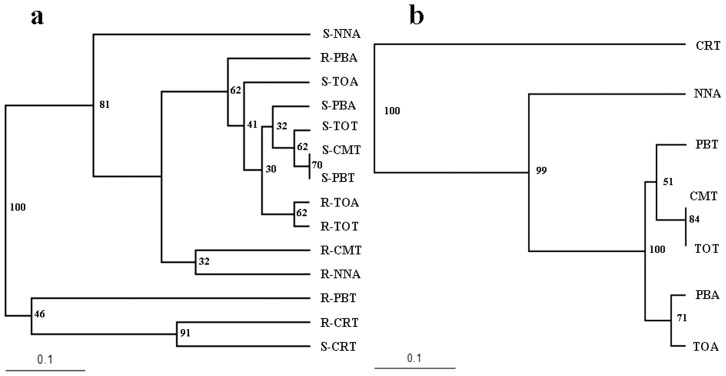
Cluster analysis of terminal restriction fragment length polymorphism patterns from AM fungal communities associated with *Aquilaria crassna* (A) and *Tectona grandis* (T); a) TRFs patterns in roots (R-) and rhizosphere soils (S-) and b) TRFs patterns in five sites (CM: Chiang Mai, CR: Chiang Rai, NN: Nakhon-Nayok, PB: Phetchabun and TO: Thai Orchid Labs). The unweighted pair-group average (UPGMA) algorithm was used to cluster patterns based on Jaccard similarities. Percentage values based on 1000 bootstrap replicates are given at each node.

The total number of different TRFs was used as a measure of AM fungal community diversity. Thirty eight TRFs were found in total for the AML3 (FAM-labelled) primer, while the NS31 (HEX-labelled) primer identified 30 TRFs. Since the AML3 primer revealed many more TRFs than the NS31 primer, only the AML3 fragments were used. Overall, in the roots and soils of *T. grandis* and *A. crassna*, we found 13 different AML3 TRFs after digestion with HinfI, 14 after digestion with Hsp92II and 11 after digestion with MboI. The mean number of TRFs in *T. grandis* root and soil samples was 5.67 and 7.67, respectively when the TRF data of the three enzymes were pooled (Figure 1). It is possible to estimate the minimum average number of AM fungi colonizing the *T. grandis* root samples by dividing the average number of TRFs by 3 (three enzymes and one labeled end) [31]. Thus, there were on average at least 1.89 fungal taxa colonizing each *T. grandis* root sample and 2.55 fungal taxa in surrounding soils, respectively. The values for *A. crassna* were at least 2.85 fungal taxa per root sample and 2.33 fungal taxa in surrounding soils. The mean number of TRFs per sample was not significantly affected by source of samples (root and soil) (*F* = 0.159, *P* = 0.693) and host plant (*F* = 3.452, *P* = 0.074) (Table S2), but there was a statistically significant effect of collecting sites (*F* = 42.77, *P*<0.01), and a significant interaction among those three factors (Table S2). The cluster analysis of TRF patterns in roots (R-) and rhizosphere soils (S-) of *A. crassna* and *T. grandis*, based on Jaccard similarities, showed no significant grouping of samples by sites and source of samples (root and soil) (Figure 2a). This suggested that the AM fungal community in roots and rhizosphere soils was almost independent in *A. crassna* (A) and *T. grandis* (T) plots. Some TRF patterns in roots and rhizosphere soils that were collected from the same site were similar, e.g. R-CRT versus S-CRT and R-TOA versus R-TOT. Combining roots and rhizosphere soils of each plant by sampling site (CM: Chiang Mai, CR: Chiang Rai, NN: Nakhon-Nayok, PB: Phetchabun and TO: Thai Orchids Lab) indicated a tendency for *T. grandis* plots to be grouped together (PBT, CMT and TOT) as well as some *A. crassna* plot samples (PBA and TOA) (Figure 2b). This suggests that the AM fungal community associated with each tree species was more similar across plots than were communities for different trees species at the same location. The response for CRT and NNA, however, does not support this.

### Occurrence of AM fungi in soils and roots of both plants

**Figure 3:**
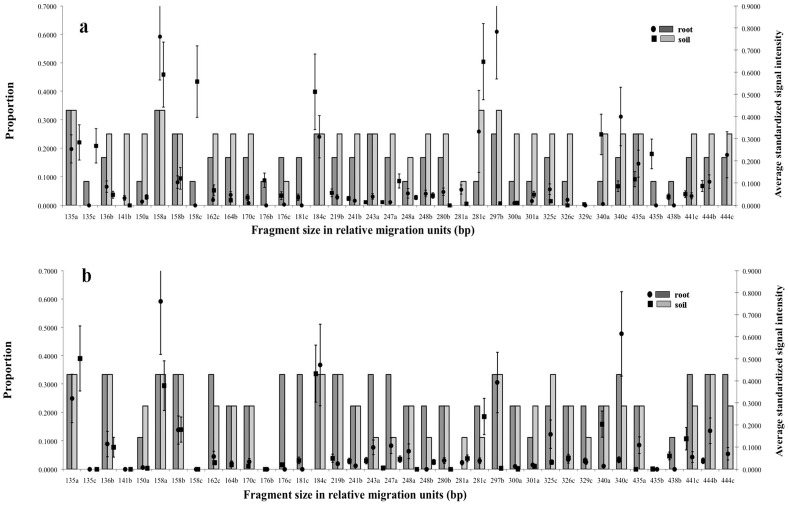
Occurrence of TRFs from roots and soils in (a) *Tectona grandis* and (b) *Aquilaria crassna*. Bars indicate the proportion of samples that yielded each TRF; dots indicate the average intensity of that fragment (± SEM) in those samples. The letters indicate the restriction enzyme involved in each fragment size, a: *MboI*, b: *HinfI* and c: *Hsp92II*.

Nearly all of the distinct TRFs (31 out of 38) were found in both host plant species (Figure 3). There were some differences in AM fungal communities between *T*. *grandis* and *A*. *crassna* because the TRF 329c (TRFs are identified by their relative mobility and a code indicating the restriction enzyme that generated them: a: MboI, b: HinfI and c: Hsp92II) was not found in *T*. *grandis*, while 5 TRFs (135c, 141b, 158c, 176b, and 435b) were not found in *A*. *crassna*. Comparison of the population in roots and soils of *T. grandis* (Fig 3a) showed that 6 TRFs (135c, 158c, 176b, 181c, 435b and 438b) were found only in roots, while 141b and 281a were only found in soils. In *A*. *crassna* (Figure 3b), TRFs 176c, 181c and 438b were only found in root samples.

### Sequence and phylogenetic analysis

Clones were selected for sequencing on the basis of HinfI and Hsp92II RFLP typing. DNA sequences of 32 selected clones were determined, 7 clones from *A. crassna* and 25 clones from *T. grandis*. Predicted TRFs from the 32 virtually digested clone sequences were compared to observed TRFs from all three restriction enzymes (Table S3). A difference in size of up to 7 nucleotides was accepted as a match, because migration in capillary electrophoresis is sequence-specific, so that mobility (in rmu) is only approximately equivalent to sequence length (in bp). All predicted TRFs were observed, and the great majority of the observed TRFs were represented in the cloned sequences.

**Figure 4:**
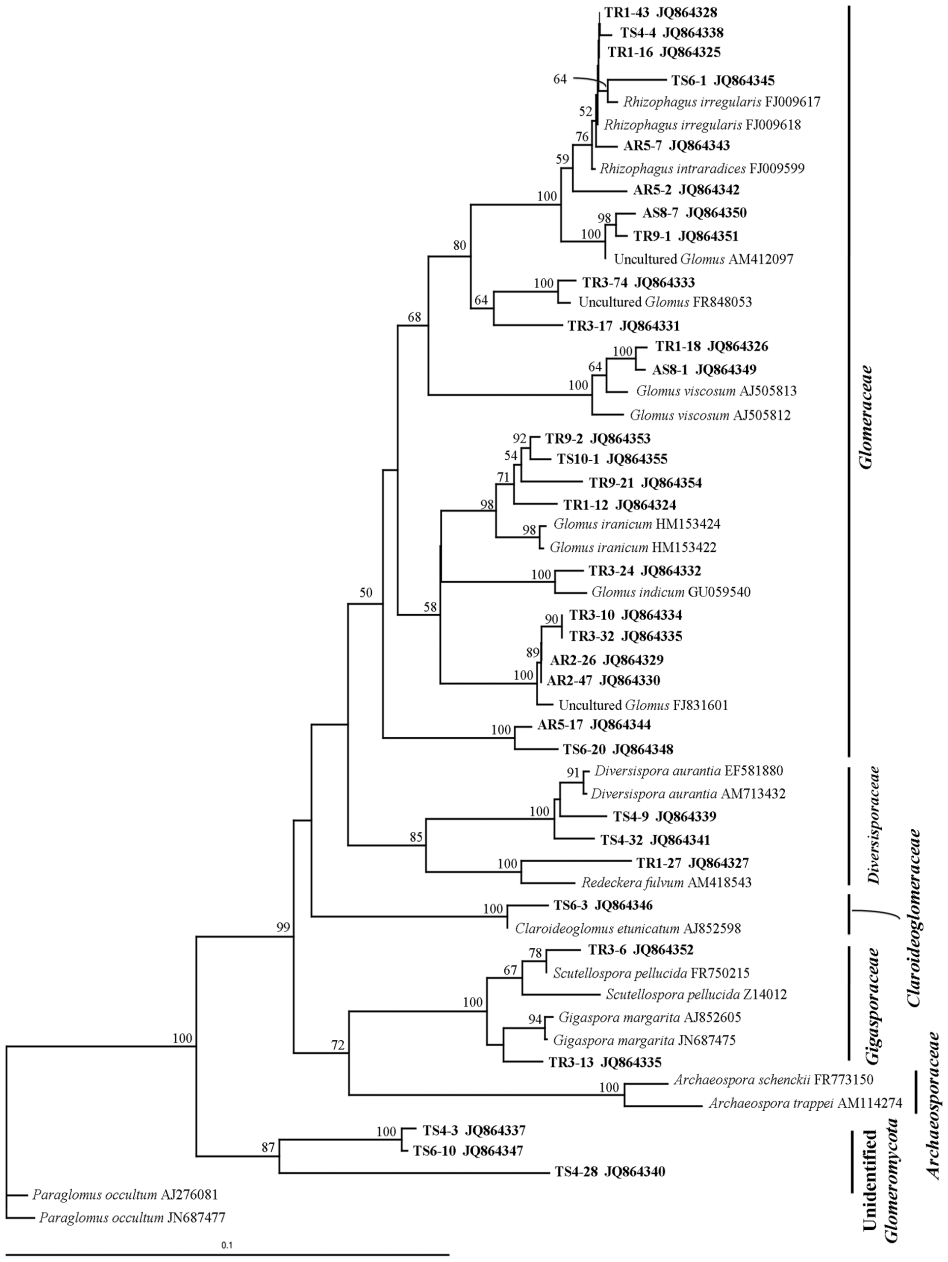
Neighbour-joining (NJ) phylogenetic tree of partial small subunit rRNA gene. Phylogeny was constructed using the region from NS31 to AML3. The percentage support values are based on 1000 bootstraps.

Our phylogenetic analysis was based on the new classification of Krüger et al. [44]. The 32 clone sequences were aligned with 23 sequences identified as closely related reference sequences in GenBank and a phylogenetic tree was constructed using the 18S rRNA gene sequences of *Paraglomus occultum* (GenBank accessions AJ276081 and JN687477) as outgroup. This indicated the presence of five AM fungal clades belonging to the families Claroideoglomeraceae, Diversisporaceae, Gigasporaceae, and Glomeraceae (Figure 4), the most frequent sequences corresponding to Glomeraceae. The subclusters contained close matches to taxa previously identified by Singh et al. [22] based on spore morphology of AM fungi in rhizosphere soils of *T. grandis*: TR1-16, TR1-43, TS4-4, AR5-7 and TS6-1 are close to *Rhizophagus intraradices* or *R. irregularis*, while TR1-27 is close to *Redeckera fulvum*. Clone sequences TS4-9 and TS4-32 are similar to *Diversispora aurantia*, while TR3-R10 is probably *Gigaspora margarita*. When sequence data are compared with individual TRFs (Table S3 and Figure 4), it is clear that individual TRFs cannot be used to identify sequence type, because many different species may generate a TRF of the same size. For example, the FAM fragment at 164b could equally well be from *G. indicum*, *Re. fulvum* or *Claroideoglomus etunicatum*.

## Discussion

This study examined the AM fungal communities of *A. crassna* and *T. grandis* plantations in Thailand. The estimated numbers of AM fungal taxa in roots and soils of *T. grandis* seedlings were 1.89 and 2.55 respectively, while in roots and soils of *A. crassna* there were 2.85 and 2.33 respectively. The AM fungal diversity was low compared with other plants. Using similar methodologies and definitions, Vandenkoornhuyse et al. [31] reported an average of 6.1 AM fungal taxa colonizing grass roots in a temperate seminatural grassland system, and 5.5 AM fungal taxa were found colonizing each *Solidago virgaurea* L. seedling root sample in low-Arctic meadow habitat [29].

Previous studies quantified the AM fungal diversity in rhizospheres of *T. grandis* and *A. crassna* mainly based on spore morphology and aimed to select efficient AM fungal isolates for growth enhancement. For example, Singh et al. [22] found an average of nine species per 100 g dry soil in a Jhum fallow site at which *T. grandis* was the dominant tree species, and most species belonging to the genus Glomus. Tamuli and Boruah [21] studied the AM fungi association of agarwood (*Aquilaria malaccensis*) plantations in Jorhat District of the Brahmaputra Valley, India. They found that the genus *Glomus* was dominant; among these *G. fasciculatum* (now known as *Rhizophagus fasciculatus*; [45]) was the most dominant followed by *G. aggregatum*. We are not aware of any information on the diversity of AM fungi on *A. crassna*. According to previous studies, we also found that most sequences belonged to the family Glomeraceae that includes *Glomus* and *Rhizophagus*. This result is consistent with previously published phylogenies [29], [39], [46]. The dominance of this family suggests that they able to survive under various agricultural conditions such as soil disturbance from plowing and cultivation and pesticide usage like that used here in the Phetchabun and Nakhon-Nayok sites. Those conditions may be unfavorable for other AM fungi. One possible reason why *Glomus* species have the ability to survive in a disturbed system is related to differences in propagation strategies [29]. Glomeraceae are capable of colonizing via fragments of mycelium, mycorrhizal root pieces, and spores, while Gigasporaceae are only capable of propagation via spores because they do not produce intra-radical vesicles: lipid-rich storage structures which allow for re-growth of hyphae from previously colonized root pieces [46], [47], [48], [49]. This difference can explain the dominance of the Glomeraceae over Gigasporaceae members in an environment with repetitive agricultural disturbance. Oehl et al. [50] revealed a clear seasonal and successional AMF sporulation dynamics and implied that different life strategies of different ecological AMF groups could be defined on the basis of diverging temporal sporulation dynamics.

This study shows that the choice of restriction enzymes (HinfI, Hsp92II, MboI) did not significantly affect AM fungal diversity found per sample. Using a combination of those three restriction enzymes could detect possible species of AM fungi in the samples, even if they resulted in similar-sized fragments. HinfI and Hsp92II were chosen in this study because they showed the highest polymorphism of cleavage sites at the extremities of the amplified DNA fragment [31]. Mummey and Rillig [39] and Wolfe et al. [51] also found that HinfI and MboI can separate different closely-related species of AM fungi identified from phylogenetic analyses. For example, *R. irregularis* and *R. intraradices* are closely related species that group in the same clade (Figure 4). Six clone sequences (TR1-16, TR1-43, TS4-4, AR5-2, AR5-7and TS6-1) that were related to both species were not completely separated using phylogenetic analysis, but virtual digesting with those three enzymes did separate them by using the combination of restriction pattern of each enzyme (Table S3). Clone sequences TR1-16, 1-43, and 6-1 grouped with *R. irregularis* and TS4-4, 5-2, 5-7 grouped with *R. intraradices*.

Some TRFs were only found in roots or only in soils, suggesting that some AM fungi may be rare in soil but produce fungal structures in roots that are rich enough for T-RFLP detection, while some were found only as spores in soils and did not colonize roots. While the majority of TRFs were associated with both *T. grandis* and *A. crassna*, some TRFs were associated with just one plant (i.e. 135c, 141b, 158c, 176b, 329c and 435b). In clustering analysis, samples from each plant species were grouped together even if they were collected from different sites. *A. crassna* samples seemed to group together, but since many AMF taxa were shared by both trees, *A. crassna* shared some AM fungal community patterns with *T. grandis* (Figure 2). Statistical analysis revealed significant effects of collecting sites and the interaction between collecting sites, host plant species and source of samples on TRFs (Table S2). Thus, specific AM taxa in roots and soils of *T. grandis* and *A. crassna* were affected by site but not affected by host plant species and source of samples (root and soil). This is in accordance with the observation of Bever et al. [52] that the host-dependence of the relative growth rates of fungal populations may play an important role in the maintenance of fungal species diversity. Previously, it has been reported that neighboring plants may have a significant impact on the AM fungal colonization and community composition of AM fungi in plant roots [34]. Although *T. grandis* at the Chiang Mai site had other *T. grandis* as closest neighbors with some negligible understory perennial plants, and at the other two sites the closest neighbors were *A. crassna*, the cluster analysis did not reveal any effect of this difference in neighbors. AM fungal community patterns in CMT were grouped with PBT and TOT sites in which weeds were controlled by agricultural management.

In conclusion, we demonstrated here that AM fungal community patterns in rhizosphere soils and roots of *T. grandis* and *A. crassna* were similar even if they were collected from different sites. AM fungal communities of *T*. *grandis* samples from different sites were similar, as were those in *A. crassna* samples. We also found that most sequences represented Glomeraceae, including *Glomus* spp. and *Rhizophagus* spp. Virtual digestion of sequences using the target sequences of the restriction enzymes HinfI, Hsp92II and MboI yielded expected fragments that mostly matched observed TRFs, linking possible AM fungal species to each TRF. Specific AM taxa in roots and soils of *A. crassna* and *T. grandis* were affected by site but were not affected by host plant species and source of samples (root and soil). Although the T-RFLP technique can provide important information about the AM fungal diversity associated with plant species of interest, trap cultures and cultured spores from the field site are still important in order to assess the ability of the AM fungi to enhance the growth of the plants, and to provide effective candidates for inoculum production targeted for these economically important tree species.

## Supporting Information

Table S1 **Correlation matrix of soil factors and terminal restriction fragments (TRFs) of study areas in wet season (July 2010) which soils were sampled.**
(DOC)

Table S2 **Summary of two-way analysis of variance for main and interaction effects of host plants (** ***Aquilaria crassna*** **and** ***Tectona grandis*** **), sites, and source of samples (root and soil) on AM fungal community diversity measured as the number of different TRFs per sample.** Significant P-values are shown in bold.
(DOC)

Table S3 **Clone sequences and TRFs derived from roots and rhizosphere soils of** ***T. grandis*** **and** ***A. crassna*** **.** Values in bold indicate TRFs that match the sizes of virtual digest fragments (with differences ranging from 0 to 7 bp).
(DOC)
