# Supplementary material for: Characterization of Arbuscular Mycorrhizal Fungus Communities of Aquilaria crassna and Tectona grandis Roots and Soils in Thailand Plantations
Source: PLoS One. 2014 Nov 14;9(11):e112591. doi: 10.1371/journal.pone.0112591 (PMC4232412; doi:10.1371/journal.pone.0112591)
Supplement: Table S1 — Correlation matrix of soil factors and terminal restriction fragments (TRFs) of study areas in wet season (July 2010) which soils were sampled. (DOC) [file pone.0112591.s001.doc]

**Table S1 Correlation matrix of soil factors and terminal restriction fragments (TRFs) of study areas in wet season (July 2010) which soils were sampled.**

| **Factors^a^** | **Nitrogen** | **Phosphorus** | **Potassium** | **OM** | **pH** | **EC** |
| --- | --- | --- | --- | --- | --- | --- |
| Nitrogen | - | - | - | - | - | - |
|  | - | - | - | - | - | - |
| Phosphorus | 0.021 | - | - | - | - | - |
|  | *P* = 0.894 | - | - | - | - | - |
| Potassium | *0.332* | - 0.076 | - | - | - | - |
|  | *P = 0.032* | *P* = 0.631 | - | - | - | - |
| OM | **0.406** | - 0.046 | 0.253 | - | - | - |
|  | ***P* = 0.008** | *P* = 0.773 | *P* = 0.106 | - | - | - |
| pH | 0.239 | **0.692** | - 0.231 | 0.018 | - | - |
|  | *P* = 0.128 | ***P* = 0.000** | *P* = 0.142 | *P* = 0.909 | - | - |
| EC | **0.402** | - 0.060 | **0.411** | - 0.060 | 0.208 | - |
|  | ***P* = 0.008** | *P* = 0.704 | ***P* = 0.007** | *P* = 0.704 | *P* = 0.186 | - |
| TRFs | 0.208 | **0.538** | 0.133 | **0.412** | **0.486** | 0.051 |
|  | *P* = 0.187 | ***P* = 0.000** | *P* = 0.401 | ***P* = 0.006** | ***P* = 0.001** | *P* = 0.746 |

^a^Factors: EC = electrical conductivity; OM = organic matter; TRFs = terminal restriction fragments.

Bold values, *P <* 0.01; italic values, *P <* 0.05.
