# Supplementary material for: Characterization of Arbuscular Mycorrhizal Fungus Communities of Aquilaria crassna and Tectona grandis Roots and Soils in Thailand Plantations
Source: PLoS One. 2014 Nov 14;9(11):e112591. doi: 10.1371/journal.pone.0112591 (PMC4232412; doi:10.1371/journal.pone.0112591)
Supplement: Table S2 — Summary of two-way analysis of variance for main and interaction effects of host plants ( Aquilaria crassna and Tectona grandis ), sites, and source of samples (root and soil) on AM fungal community diversity measured as the number of different TRFs per sample. Significant P-values are shown in bold. (DOC) [file pone.0112591.s002.doc]

**Table S2 Summary of two-way analysis of variance for main and interaction effects of host plants (*Aquilaria crassna* and *Tectona grandis*), sites, and source of samples (root and soil) on AM fungal community diversity measured as the number of different TRFs per sample. Significant P-values are shown in bold.**

| **Factor** | **d.f.** | ***F*** | ***P*** |
| --- | --- | --- | --- |
| **Host** | 1 | 3.452 | 0.074 |
| **Site** | 4 | 42.777 | **0.000** |
| **Source** | 1 | 0.159 | 0.693 |
| **Host × Site** | 1 | 7.767 | **0.009** |
| **Host × Source** | 1 | 13.808 | **0.001** |
| **Site × Source** | 4 | 4.037 | **0.010** |
| **Host × Site × Source** | 1 | 7.767 | **0.009** |
