# Supplementary material for: Characterization of Arbuscular Mycorrhizal Fungus Communities of Aquilaria crassna and Tectona grandis Roots and Soils in Thailand Plantations
Source: PLoS One. 2014 Nov 14;9(11):e112591. doi: 10.1371/journal.pone.0112591 (PMC4232412; doi:10.1371/journal.pone.0112591)
Supplement: Table S3 — Clone sequences and TRFs derived from roots and rhizosphere soils of T. grandis and A. crassna . Values in bold indicate TRFs that match the sizes of virtual digest fragments (with differences ranging from 0 to 7 bp). (DOC) [file pone.0112591.s003.doc]

**Table S3** Clone sequences and TRFs derived from roots and rhizosphere soils of *T. grandis* and *A. crassna*. Values in bold indicate TRFs that match the sizes of virtual digest fragments (with differences ranging from 0 to 7 bp).

| **Clone sequence** | **Virtual digest fragments (bp)** | | | | | | **Closest observed TRFs (rmu)** | | | | | | **AMF clade** |
| --- | --- | --- | --- | --- | --- | --- | --- | --- | --- | --- | --- | --- | --- |
|  | ***HinfI*** | | ***Hsp92II*** | | ***MboI*** | | ***HinfI*** | | ***Hsp92II*** | | ***MboI*** | |  |
|  | **F^a^** | **R^b^** | **F** | **R** | **F** | **R** | **F** | **R** | **F** | **R** | **F** | **R** |  |
| TR1-16 | 142 | 309 | 117 | 191 | 280 | 7 | **141** | 299 | **116** | **187** | **280** | ×^c^ | *Glomeraceae* |
| TR1-43 | 142 | 308 | 117 | 191 | 280 | 6 | **141** | 299 | **116** | **187** | **280** | × | *Glomeraceae* |
| TS6-1 | 142 | 309 | 117 | 191 | 280 | 6 | **141** | 300 | **116** | **193** | **280** | × | *Glomeraceae* |
| TS4-4 | 303 | 148 | 193 | 115 | 164 | 287 | **300** | **141** | **187** | **115** |  | **280** | *Glomeraceae* |
| AR5-2 | 303 | 16 | 193 | 258 | 164 | 287 | **300** | × | 184 |  | **158** |  | *Glomeraceae* |
| AR5-7 | 303 | 148 | 193 | 115 | 164 | 287 | **300** | **147** | 184 | **113** | **158** |  | *Glomeraceae* |
| AS8-7 | 142 | 309 | 117 | 191 | 132 | 6 | **141** | 300 | **116** | **187** | **130** | × | *Glomeraceae* |
| TR9-1 | 303 | 148 | 193 | 115 | 164 | 138 | **300** |  | **196** |  |  | **135** | *Glomeraceae* |
| TR3-17 |  |  | 192 | 168 | 164 | 105 |  |  |  | **170** | **158** |  | *Glomeraceae* |
| TR3-74 | 191 | 260 | 169 | 191 | 280 | 6 | **190** | 250 |  | **184** | **280** | × | *Glomeraceae* |
| TR1-18 |  |  | 193 | 115 | 141 | 104 |  |  | **190** | **115** | **141** | **105** | *Glomeraceae* |
| AS8-1 |  |  | 117 | 191 | 98 | 6 |  |  | **116** | **187** | **96** | × | *Glomeraceae* |
| TR9-2 | 164 | 196 | 192 | 164 | 141 | 310 | **160** | 187 | **190** | **160** | **135** |  | *Glomeraceae* |
| TR9-21 | 164 | 17 | 193 | 164 | 141 | 310 | **160** | × | **190** | **160** | **135** |  | *Glomeraceae* |
| TS10-1 | 164 | 196 | 193 | 164 | 141 | 310 | **165** | **199** | 184 | **162** | **141** | 301 | *Glomeraceae* |
| TR1-12 | 163 | 287 | 192 | 165 | 140 | 310 | **161** | **280** | **190** | **165** | **141** | **307** | *Glomeraceae* |
| TR3-24 | 190 | 170 | 258 | 193 | 302 | 149 | **190** | **164** |  | 184 | **303** | 134 | *Glomeraceae* |
| AR2-26 | 141 | 170 | 258 | 193 | 98 | 7 | **141** | **170** | **260** | **193** | **96** | × | *Glomeraceae* |
| AR2-47 | 142 | 309 | 191 | 260 |  |  | **141** | **297** | **187** | **258** |  |  | *Glomeraceae* |
| TR3-10 | 141 | 170 | 258 | 193 | 98 | 7 | **141** | **164** |  | 184 | **96** | × | *Glomeraceae* |
| TR3-32 | 141 | 170 | 258 | 193 | 98 | 7 | **141** | **164** |  | 184 | **96** | × | *Glomeraceae* |
| AR5-17 | 191 | 260 | 259 | 192 | 280 | 6 | **190** | **260** | **259** | **187** | **280** | × | *Glomeraceae* |
| TS6-20 | 254 | 197 | 193 | 258 | 141 | 286 |  | **199** | **187** | **258** | **141** | **280** | *Glomeraceae* |
| TS4-9 | 142 | 309 | 170 | 190 | 305 | 4 | **141** | 300 | **169** | **188** | **305** | × | *Diversisporaceae* |
| TS4-32 | 305 | 146 | 194 | 166 | 141 | 310 | **300** | **141** | **187** | **165** | **141** | **307** | *Diversisporaceae* |
| TR1-27 | 254 | 164 | 193 | 167 | 141 | 310 |  | **164** | **190** | **165** | **141** | **307** | *Diversisporaceae* |
| TR3-6 | 142 | 309 | 191 | 260 |  |  | **141** | 297 |  |  |  |  | *Gigasporaceae* |
| TR3-13 | 158 | 293 | 168 | 192 | 302 | 7 |  | **297** |  | 184 | **303** | × | *Gigasporaceae* |
| TS6-3 | 287 | 164 | 264 | 163 | 309 | 142 | **286** | **164** | **264** | **163** | **309** | **140** | *Claroideoglomeraceae* |
| TS4-3 |  |  | 264 | 187 | 200 | 251 |  |  | **264** | **187** | **197** | **247** | Unidentified *Glomeromycota* |
| TS4-28 |  |  | 263 | 188 |  |  |  |  | **264** | **188** |  |  | Unidentified *Glomeromycota* |
| TS6-10 |  |  | 264 | 187 | 200 | 251 |  |  | **264** | **187** | **201** | **247** | Unidentified *Glomeromycota* |

^a^ F: NS31 (forward) TRF, ^b^ R: AML3 (reverse) TRF, ^c^ ×: fragments that would be beyond the detection range (50-450 bp).
